# Biomimetic substrate control of cellular mechanotransduction

**DOI:** 10.1186/s40824-016-0059-1

**Published:** 2016-04-29

**Authors:** Mohammad Nahid Andalib, Yuris Dzenis, Henry J. Donahue, Jung Yul Lim

**Affiliations:** Department of Mechanical and Materials Engineering, University of Nebraska-Lincoln, W317.3 Nebraska Hall, Lincoln, NE 68588-0526 USA; Department of Biomedical Engineering, Virginia Commonwealth University, 401 West Main Street, P.O. Box 843067, Richmond, VA 23284-3067 USA

**Keywords:** Biomimetic substrate, Mechanical stimulation, Mechanotransduction, Functional tissue engineering

## Abstract

Extracellular mechanophysical signals from both static substrate cue and dynamic mechanical loading have strong potential to regulate cell functions. Most of the studies have adopted either static or dynamic cue and shown that each cue can regulate cell adhesion, spreading, migration, proliferation, lineage commitment, and differentiation. However, there is limited information on the integrative control of cell functions by the static and dynamic mechanophysical signals. For example, a majority of dynamic loading studies have tested mechanical stimulation of cells utilizing cultures on flat surfaces without any surface modification. While these approaches have provided significant information on cell mechanotransduction, obtained outcomes may not correctly recapitulate complex cellular mechanosensing milieus in vivo. Several pioneering studies documented cellular response to mechanical stimulations upon cultures with biomimetic substrate modifications. In this min-review, we will highlight key findings on the integrative role of substrate cue (topographic, geometric, etc.) and mechanical stimulation (stretch, fluid shear) in modulating cell function and fate. The integrative approaches, though not fully established yet, will help properly understand cell mechanotransduction under biomimetic mechanophysical environments. This may further lead to advanced functional tissue engineering and regenerative medicine protocols.

## Background

Mechanical loading plays a vital role in tissue homeostasis [1, 2]. Also for the regeneration of a more biomechanically-competent tissue constructs, physiologically relevant, controlled mechanical loading is critically needed. A wide variety of cell functions such as orientation, migration, proliferation, lineage commitment, and differentiation has been shown to respond to different modes of mechanical loading, as in our group’s reports [3–6]. Many other studies have also reported that mechanical loading, such as stretch, fluid shear, compression, and others, could contribute to successful regeneration of mechanically functional tissues such as cardiac, muscle, vasculature, ligament, tendon, bone, and so on [7–12]. Different loading mode can be a purpose-specific regulator of cellular systems, e.g., mechanical strain contributed to mesenchymal stem cell (MSC) differentiation into smooth muscle cells and chondrocytes [13, 14] while fluid shear stress could induce their differentiation towards endothelial cells [15]. To take advantage of mechanical loading for the functional tissue engineering, several types of bioreactors have been developed that provide different loading modes such as shear flow, tension, torsion, or combination of these [16].

In addition to dynamic mechanical loading, static mechanophysical signals given by the cell culture substrates also have a strong potential to affect cell function and fate. It has long been established that changes in substrate topographic and geometric features (e.g., isotropic and anisotropic topographic patterns, micro and nanoscale surface patterning, etc.) can direct cellular adhesion, spreading, orientation, alignment, and migration, and via this affect downstream cell behaviors including cell survival and apoptosis, cell-cell interaction, lineage specification, and terminal differentiation (see more details in our previous review [17]). Significant developments in substrate fabrication techniques have allowed the investigation of cell behaviors on substrates with a more biomimetic characteristic. These include photo- and electron beam lithography, soft lithography, nanoimprint lithography, electrospinning, polymer demixing, 3D printing, etc. [17–22].

Although each mechanical stimulation and substrate induction are well recognized as described above, little is known in regard to their integrative control of cellular functions. It is true that conventional cell mechanotransduction studies have dealt with cells cultured on plain surfaces, for example, mechanical stretching of cells seeded on elastic, flat membranes or fluid flow of cells seeded on glass slides. While these approaches provide advantages in assessing cellular mechanotransduction pathways via allowing easiness in imaging and RNA and protein sample collection, tests on simple flat surfaces would not necessarily recapitulate complex cellular mechanosensing environments in vivo, thus potentially depreciating the usefulness of the identified molecular mechanisms. Several studies reported pioneering data on cellular responses to mechanical stimulations upon cultures with biomimetic substrate modifications. In this mini-review, rather than in-depth technical or mathematical description of various mechanical cell stimulation methods or substrate modification techniques, we will highlight key findings on cellular responses to mechanical stimuli on biomimetically modified substrates. Specifically, how cell sensing of and response to mechanical stretch and fluid shear can be modulated via biomimetic substrate cultures will be focused. Understanding the crosstalk between engineered substrate and mechanical loading in affecting cellular mechanotransduction under correctly combined conditions could be of benefit for both biomaterials science and mechanobiology. This approach will further advance the theories and applications of functional tissue engineering and regenerative medicine.

## Review: mechanical cell stimulation on biomimetic substrates

### Mechanical stretching of cells on biomimetic substrates

Cells in vivo are often exposed to aligned extracellular matrix (ECM) architectures and respond to them by orienting and elongating themselves along the anisotropic matrix direction, i.e., contact guidance [17]. Various synthetic ridge and groove topographies have been produced to mimic anisotropic in vivo architectures, and studies using these synthetic topographies demonstrated that contact-guided cell alignment could be replicated in vitro. On the mechanical loading side, studies have shown that in response to mechanical stretching the cells actually aligned perpendicular to the stretch direction [23–25]. A potential cellular mechanism of the perpendicular cell orientation to the stretch, e.g., to relieve cellular tension under stretch loading, is described in our review [25]. Combining the two results, i.e., cell alignments along the groove direction and perpendicular to the stretch direction, it would be interesting to test how cells will be aligned under two superposed cues. The design will include the case in which the stretch is applied to the direction parallel or traverse to the anisotropic groove. For this, stretchable microgroove topographies were fabricated by using elastic substrates, e.g., custom-made silicone dishes [26, 27]. It was observed in these studies that cell alignment may be more affected by topographic guidance relative to stretch signal. When fibroblasts cultured on microgrooved substrates were subjected to cyclic uniaxial stretching, the cells did not alter their contact-guided alignment by the additional stretch cue regardless of the stretching direction. Another study also concluded that substrate control may play a primary role in cell shaping. In the study using two different stretchable topographies, a 10 μm wide square-groove and 40 μm wide V-groove, fibroblasts primarily adjusted their orientation according to the anisotropic substrates while stretching only played a secondary role [28].

**Fig. 1:**
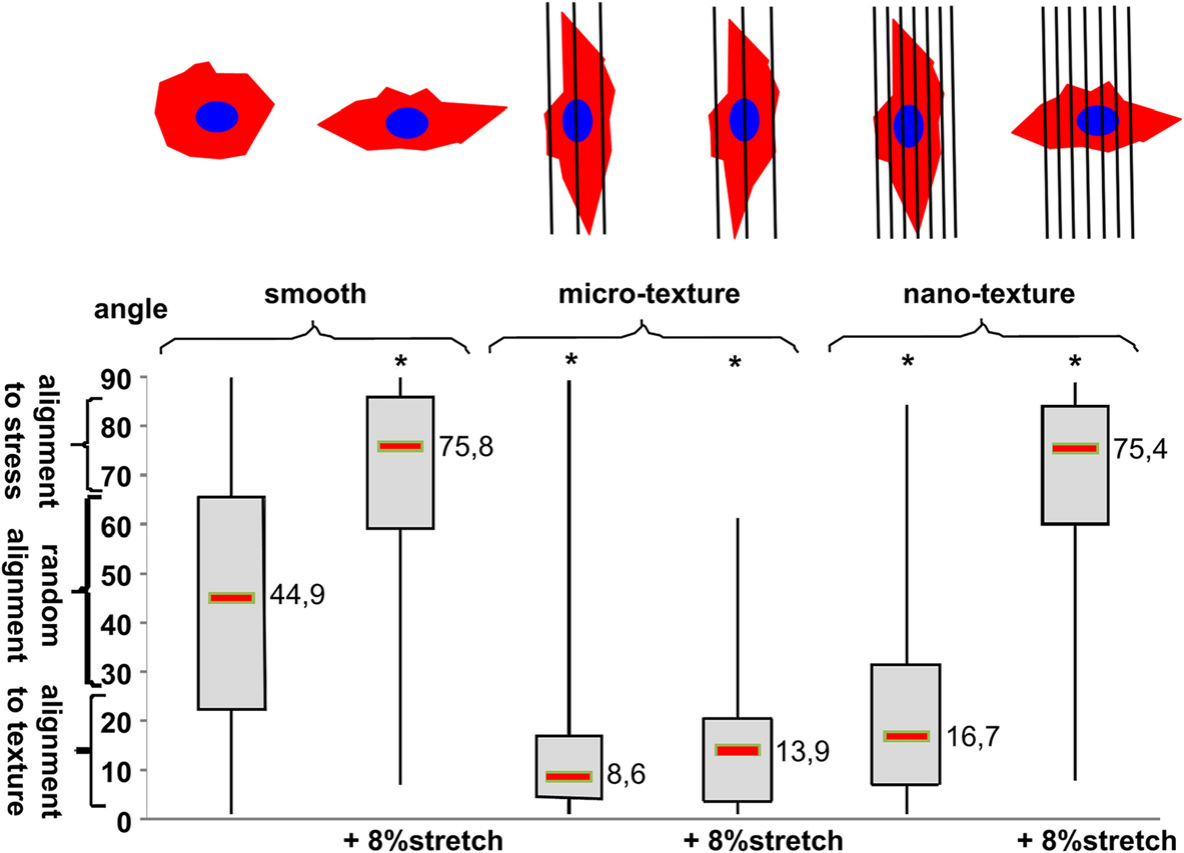
When subjected to stretching, osteoblasts cultured on nanogrooves lose their alignment along the groove direction. Box-Whisker plot of the cell alignment on varying substrates (smooth control, microgroove, nanogroove) without or with stretching. Cell alignment on the microgrooves was not affected by 8 % stretching, while on nanogrooves cell alignment was lost due to the stretching. *: *p* < 0.001 compared with the smooth control (reprinted from Prodanov et al. [30] with permission from Elsevier)

In a potential competitive control of cellular orientation by anisotropic substrate and mechanical stretch, there may exist criteria for groove dimension to determine the competition. In the study by Houtchens et al. [29], vascular smooth muscle cells showed limited orientation response when stretch direction was parallel to the microgrooves, but exhibited enhanced cell alignment on grooves when stretch was applied perpendicular to the grooves. Further, cells better aligned in response to stretch on either small (15 μm) or large (70 μm) width grooves compared with intermediate width (40 μm), suggesting an existence of the optimal groove dimension to increase cellular mechanosensitivity to stretch signal. The comparison was further extended to nanotopographies in the study of Prodanov et al. [30], which tested nanogrooves (300 nm width, 600 nm pitch, 150 nm depth) vs. microgrooves (1 μm width, 2 μm pitch, 500 nm depth). They showed that osteoblasts on smooth control surfaces showed perpendicular orientation to the stretch, as reported for other cells [23–25]. Osteoblasts seeded on microgrooves displayed contact guidance and did not change their alignment by the longitudinal stretch (along the grooves), similar to the microgroove data described above implying the primary role of grooves [26–28]. However, interestingly, cells cultured on nanogrooves lost their alignment along the groove direction when subjected to 8 % longitudinal stretching, thus exhibiting perpendicular orientation with respect to the nanogroove direction (Fig. 1). This indicated that the stretch signal could overcome the substrate guidance for the case of nanoscale grooves. Combined, contact guidance from anisotropic substrates may compete with added stretch signals and the outcomes need to be carefully viewed depending on the scale of the grooves.

**Fig. 2:**
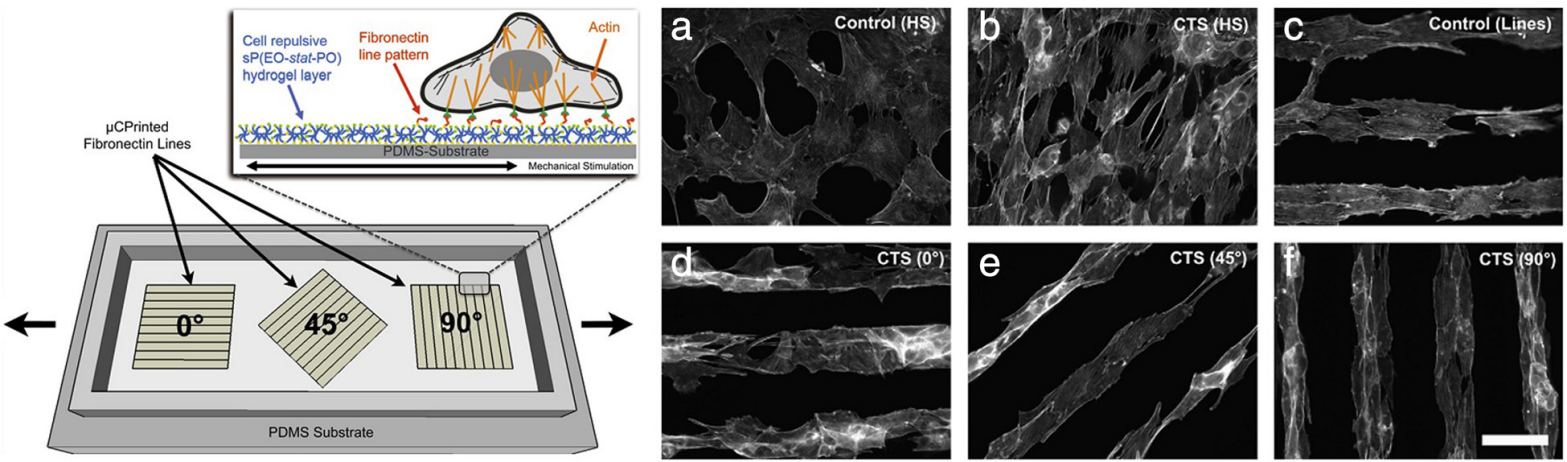
Cyclic tensile stretching parallel to the micro-patterned cell lines (0° stretch) results in irregular myoblast orientation. (*Left*) The cell stretching device in which cell adhesive lines were oriented at 0°, 45°, and 90° to the stretch direction. (*Right*) **a**-**f** Actin stress fiber orientation in C2C12 myoblasts. Unstretched controls on HS (homogenous surface) and line patterns are shown. Cells exposed to cyclic tensile stretching (CTS) on HS are shown (stretch direction is horizontal). CTS applied for cell line patterns at varying stretch angles are also shown (reprinted from Ahmed et al. [31] with permission from Elsevier)

The substrate-stretch combined control was also tested with chemically micropatterned surfaces. Micro-contact printing of cell-adhesive ligands is another established way to achieve preferred cell alignment and elongation to a certain direction [17]. Ahmed et al. [31] developed a cellular micropatterning system which can be subjected to a stretching motion at varying angles from the cell alignment direction (Fig. 2). The cellular orientation perpendicular to the stretch direction was confirmed again for C2C12 myoblasts stretched on non-patterned surfaces. When the myoblasts confined within the micro-contact printed fibronectin lines were subjected to the stretches, changes in actin stress fiber orientations could be detected. Stretch applied parallel to the micro-patterned lines (0° stretching) rendered cells to orient irregularly and as a result actin stress fibers were oblique to the stretch direction. On the other hand, stretches applied at 45° and 90° to the patterned lines produced actin stress fiber orientation angles comparable to the stretch angles. Data clearly add information on the correlation between the stretch direction and the imposed cell alignment angle before stretch in determining cellular stretch sensitivity.

**Fig. 3:**
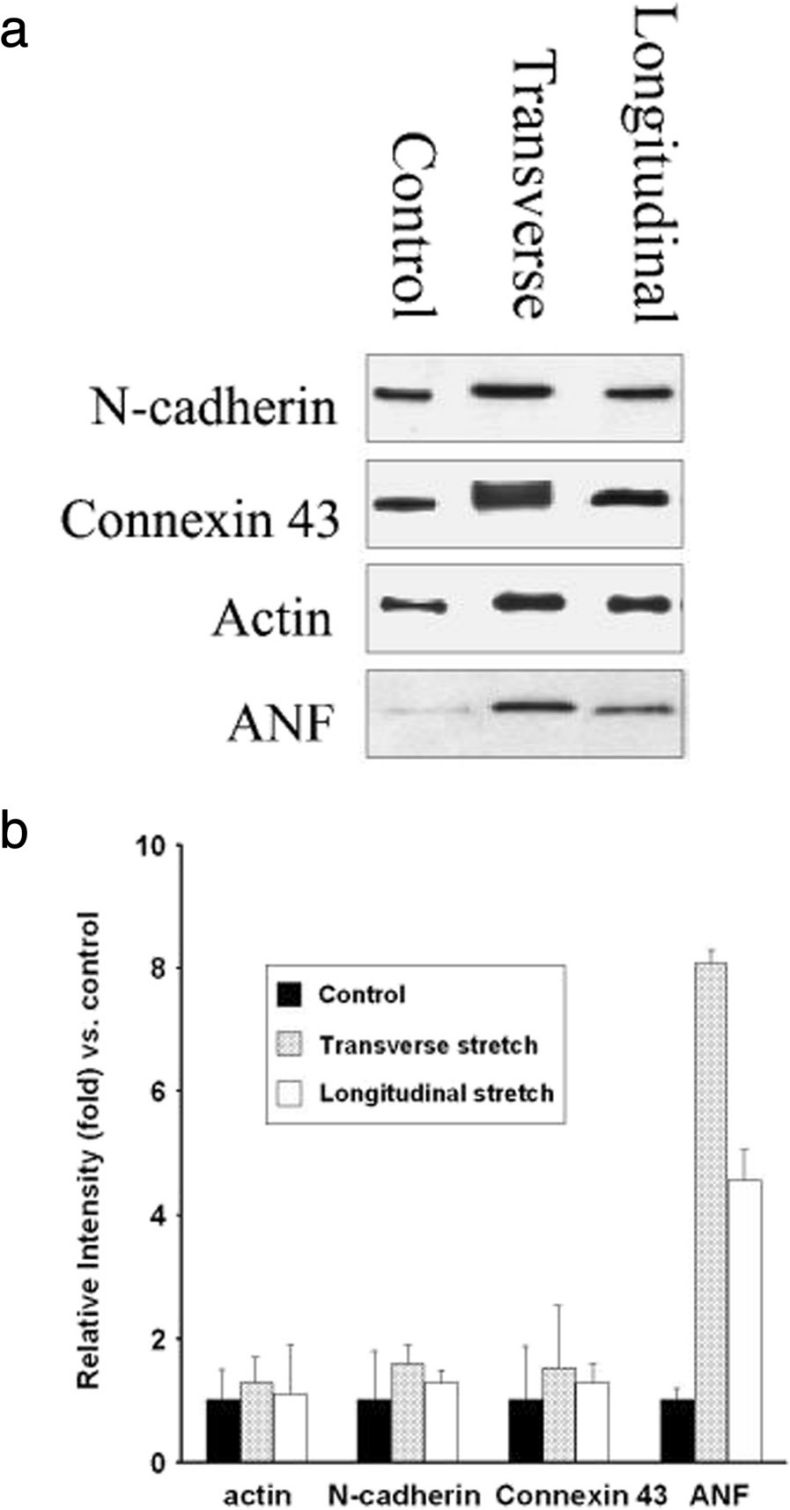
Stretching transverse to the cell patterning direction increases the expressions of atrial natriuretic factor and cell-cell junction molecules in myocytes. **a** Western blot of N-cadherin, connexin 43, and atrial natriuretic factor (ANF) of myocytes patterned and exposed to stretches. **b** Immunoblot intensity compared with control (reprinted from Gopalan et al. [35] with permission from Wiley)

Gene expression and molecular mechanosensors potentially relevant to the changes in cell orientation under the substrate-stretch combined signal were examined. Park et al. [32] showed that longitudinal cyclic stretching along the groove direction made ligament fibroblasts align away form the microgroove patterns, indicating that stretch could be more influential in their case (similar to the nanogroove case in Fig. 1). They also showed that MGP, GADD45A, UNC5B, TGFB1, COL4A1, and COL4A2 genes, which play a crucial role in cell growth and apoptosis, differentiation, and homeostasis, were upregualted by the microgroove and stretch combined stimuli. Another study showed that cyclic stretching of human tendon fibroblasts on microgrooved silicone membranes increased the activity of inflammatory prostaglandin E2 (PGE2), a known tendinitis mediator, and related cyclooxygenase (COX) sensor [33]. This suggested that inflammatory response of the fibroblasts may depend on both substrate and stretch stimuli. The mechanosensor related to cell nuclei and chromatin remodeling may also be affected by substrate and mechanical cues. In a study of MSCs cultured on microgrooves and exposed to stretches [34], only stretch perpendicular to the microgrooves resulted in a decrease in the histone deacetylase activity. This change accompanied alteration in the nuclear shape. It was proposed that lamin, an inner nuclear protein, could play a role as a mechanosensor governing the observed MSC responses. In the study by Gopalan et al. [35], cardiac myocytes were micropatterned and statically stretched either parallel or transverse to the patterning direction. Again, only the stretch transverse to the patterning direction could increase the accumulation of myofibrils and the expressions of atrial natriuretic factor (ANF) and cell-cell junction molecules such as connexin 43 (Cx43) gap junction and N-cadherin adherens junction (Fig. 3). Combined, various molecular sensors, intracellular and intercellular, may be affected by the substrate-stretch cues but to a different degree depending on the stretch direction.

In addition to microgrooved substrates, aligned electrospun nanofibers may also provide cell alignment signal [20]. Utilizing this capability, cells seeded on nanofibers have also been tested for the stretch sensitivity [36–38]. The evolution of intracellular calcium concentration ([Ca^2+^]_i_), one of the markers of cellular mechano-responsiveness, was assessed for meniscus fibrochondrocytes (MFCs) cultured on aligned nanofibers and exposed to longitudinal stretch (along the aligned nanofibers) [37]. The [Ca^2+^]_i_ in response to stretch on aligned nanofibers was substantially different from that in the native meniscus tissue, e.g., significantly more frequent Ca^2+^ peaks on nanofibers than the native tissue. Further, taking advantage of nanofibers that can be used as tissue engineering scaffolds, co-control of MSC differentiation by substrate (nanofiber) and mechanical stretch was attempted [38]. The differentiation of MSCs to ligament fibroblasts could be accomplished when MSCs were cultured on aligned nanofibers and co-stimulated with longitudinal stretching. However, MSCs seeded on random nanofibers failed to undergo such differentiation even in the presence of stretch.

Other than anisotropic substrate cues (grooves, lane micropatterns, aligned nanofibers, etc.) as described above, isotropically modified substrates have also been used for testing cellular sensitivity to the stretch signal. Isotropic substrate modifications, e.g., randomly or uniformly distributed topographic features (islands, pits, etc.) both at micro and nanoscale, have been widely utilized as another biomimetic platform for cell culture [17]. However, only a few studies attempted their integration with mechanical stretch. For instance, a combined effect of uniformly distributed microisland surfaces and mechanical stretch on cellular neurogenesis was examined [39]. Microisland textures were found to promote neurite outgrowth under low or static stretch condition, but interestingly the effect was decreased at high strains. In a study using randomly roughened stainless steel surfaces, cultured human MSCs could be exposed to mechanical forces via an electromagnet system that uses magnetic collagen-coated particles [40]. MSCs cultured on rough surfaces showed a rapid upregulation in phosphorylated focal adhesion kinase (p-FAK at Tyr-397) by the mechanical stimuli, which was not observed on smooth surfaces. This suggests that FAK activation may be required for MSC mechanical sensing and functioning on metallic implants with rough surfaces.

### Fluid shear stimulation of cells on biomimetic substrates

**Fig. 4:**
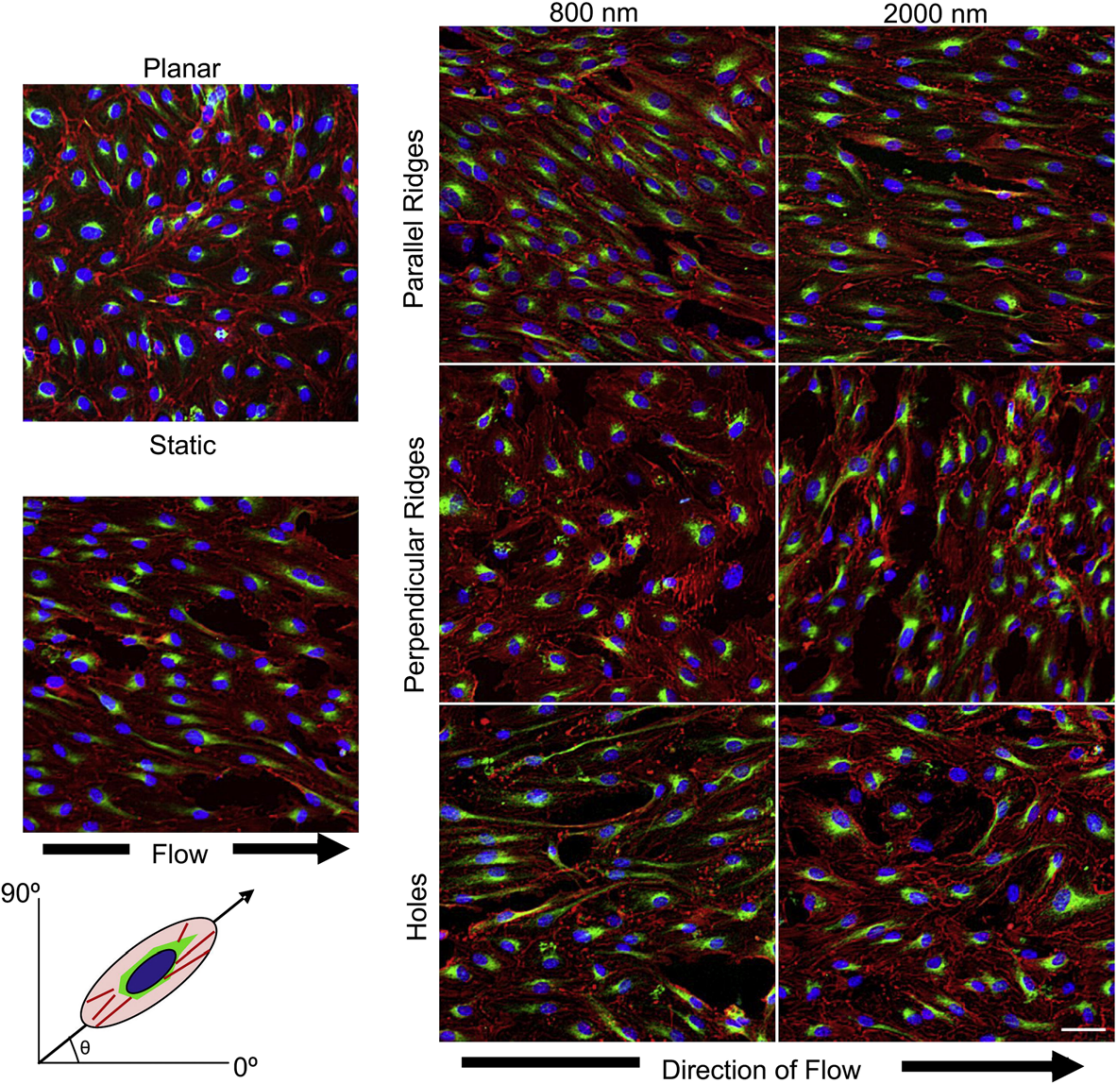
Fluid shear applied parallel to the grooves produces a synergistic effect on endothelial cell orientation, while perpendicular flow results in disorganized cell orientation. Cells were either cultured on planar control or groove and hole topographies with varying dimensions (800 and 2000 nm) and exposed to steady fluid flow at 20 dynes/cm^2^ (reprinted from Morgan et al. [41] with permission from Elsevier)

In substrate-stretch combination cases described above, it was tested how cells will align under the two stimuli, i.e., anisotropic substrate to induce contact guidance vs. mechanical stretch to provide perpendicular cell orientation. Similar tests were conducted for the substrate-fluid shear integrative control. The goal was to determine whether fluid shear induction of cell alignment along the flow direction (unlike the stretch case giving perpendicular orientation) will produce a synergistic or competitive effect with contact guidance. In the study by Morgan et al. [41], endothelial cells showed alignment along the flow direction relative to stochastic cell orientation on planar surfaces without flow (Fig. 4). Fluid shear applied parallel to the grooves produced synergistic impact on cell orientation along the grooves, while perpendicular flow resulted in an antagonistic effect to disorganize cell orientation.

**Fig. 5:**
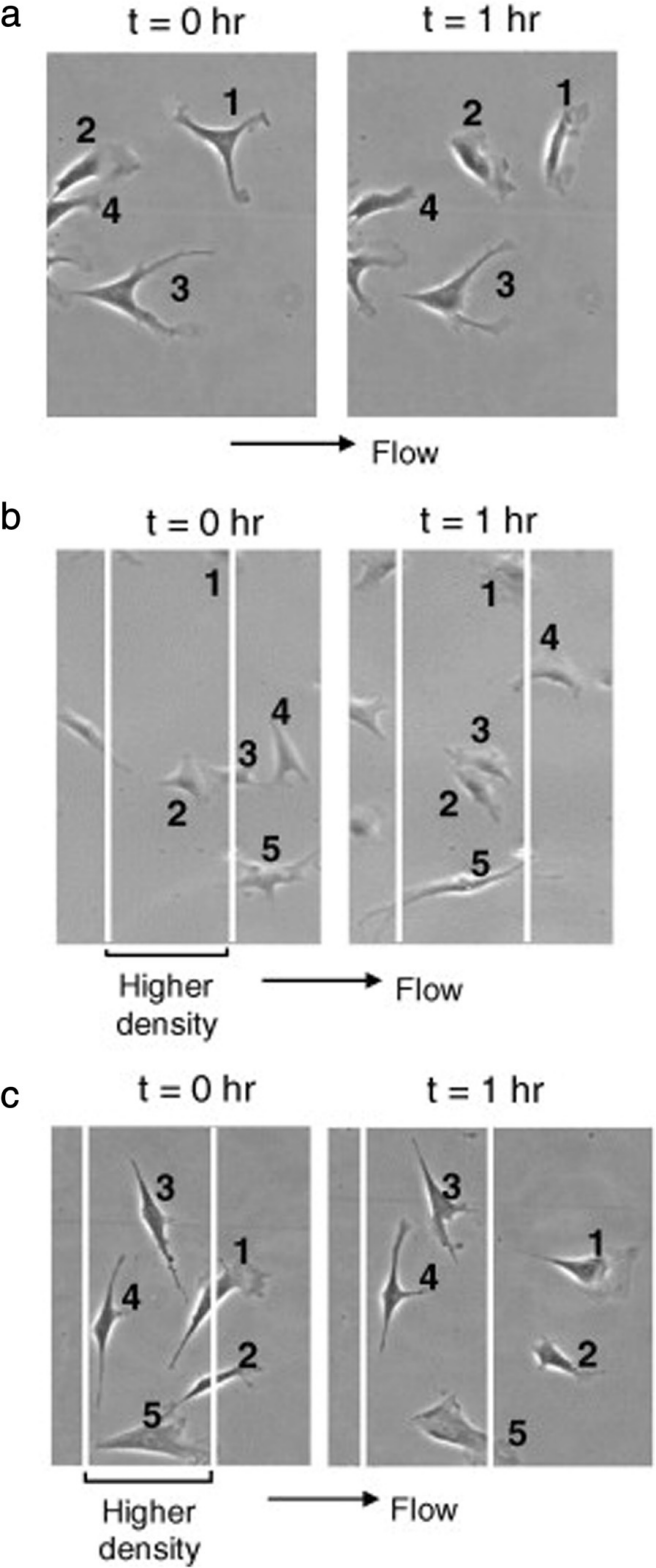
Higher fluid shear stress induces endothelial cell migration against the haptotaxis. **a** Fluid shear at 6 dyne/cm^2^ induced endothelial cell migration. **b** Lower shear stress (2 dyne/cm^2^) did not reverse haptotaxis (cell migration toward the collagen lane pattern). **c** Higher shear stress (6 dyne/cm^2^) could induce cell migration away from the collagen pattern (reprinted from Hsu et al. [43] with permission from Elsevier)

A few other substrate-fluid shear combinatory studies have focused on cell migration behaviors, mostly aiming to determine if substrate-mediated cell migration can be overcome by fluid shear induction. For endothelial cell migration on poly(dimethylsiloxane) microgrooves under flows, both magnitude and direction of the fluid shear had effects to guide cell migration [42]. Endothelial cells typically migrated to the groove direction under static condition, and the migratory pattern was not altered when cells were subjected to moderate fluid shear stress (13.5 dyne/cm^2^) irrespective of the flow direction. Interestingly, if cells experienced high shear stress (58 dyne/cm^2^) transverse to the grooves, cells started to migrate in the orthogonal direction to the grooves. It is notable that even though the migration was altered due to the transverse shear stress, focal adhesions and actin filaments kept their original alignment structures along the grooves. Based on this, they concluded that the substrate cue may still be more effective in guiding endothelial migration. Another study on endothelial cell migration by Hsu et al. [43] tested the competition between haptotaxis (ECM gradient-dependent cell migration) and mechanotaxis (shear force-dependent migration). The endothelial cell migration toward a patterned collagen, i.e., haptotaxis, was not perturbed by lower shear stress (2 dyne/cm^2^) (Fig. 5). However, higher shear stress (>3 dyne/cm^2^) induced endothelial cell movement against the haptotaxis. Combined data suggest that fluid shear can compete with contact guidance or haptotaxis in affecting cell migration, but the magnitude of shear stress to overcome such effects may vary.

The potential of nanofibrous substrates to mimic ECM nanofilamentary architecture can be integrated with microfluidic platforms that can generate spatially and temporally defined flow microenvironments. The nanofiber-microfluidic integration may thus provide biomimetic cell growth environments required for regenerative medicine, as proposed and developed by Wallin et al. [44]. Another study also developed a nanofiber-microfluidic device via which MSC responses seeded on aligned nanofibers could be examined at varying fluid flow directions (0°, 45°, 90°) to the aligned nanofibers [45]. Their results suggested that MSC morphology and fate decision may depend on the fluid shear magnitude and direction to the aligned nanofibers. Specifically, when the fluid shear was perpendicular to the aligned nanofibers, it was conducive to MSC fibrochondrogenesis. On the other hand, the parallel flow allowed MSCs to show fibroblastic phenotype. In signaling pathway studies, RhoA kinase (ROCK) and yes-associated protein (YAP)/transcriptional co-activator with PDZ-binding motif (TAZ) were proposed to govern the nanofiber-fluid shear induction of MSC fibrochondrogenesis, since the differentiation was disrupted by Y-27632, a ROCK inhibitor, and the small interference RNA (siRNA) of YAP/TAZ.

Some studies on nanofiber-fluid shear combination reported potential cell detachment from the nanofibers under high shears. When the neurite outgrowth behavior of PC-12 cells was assessed using nanofibrous culture and fluid flow, higher shear stresses preferably enhanced cell alignment and thus neurite outgrowth but increased shear stress would sometimes result in the detachment of neuronal cells from nanofibers [46]. In an endothelial cell culture on electrospun nanofibers and under fluid shear, cells cultured on aligned nanofibrous scaffolds had greater resistance to detachment compared with those on random nanofibers [47]. Combined with this result, increased F-actin bundle formation and VE-cadherin expression by fluid shear on aligned nanofibers suggested that aligned topographical guidance could be an effective mean to enhance endothelial cell adhesion for functional vascular tissue engineering.

**Fig. 6:**
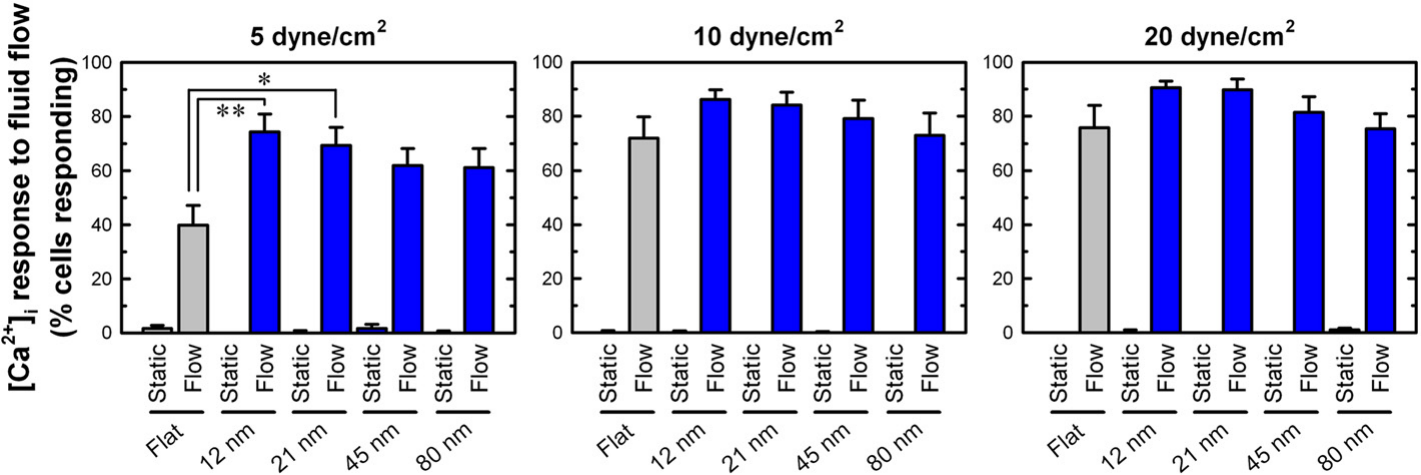
Human MSCs cultured on 12 and 21 nm high nanoislands have increased mechanosensitivity to fluid shear. The percentage of cells responding in intracellular calcium, [Ca^2+^]_i_, under fluid shear stimulation. Human MSCs were cultured on nanoisland topographies with varying island heights and flat controls and exposed to shear stresses. *: *p* < 0.05, **: *p* < 0.01 compared with flat control (reprinted from Salvi et al. [48] with permission from Elsevier)

As attempted in substrate-stretch cases, isotropic textures have also been utilized for investigating cell sensitivity to fluid shear. In our previous study [48], we tested the hypothesis that mechanosensitivity of human MSCs would be increased when cultured on randomly-distributed nanoisland topographies than on flat surfaces. It extended our previous observations under static culture that nanoisland or nanopit topographies at specific nanoisland height or nanopit depth, e.g., 10–20 nm scale, could significantly improve integrin-mediated focal adhesion, linker protein (paxillin, vinculin) expressions, FAK phosphorylation at Tyr-397, cultured osteoblastic cell modulus, and MSC fate decision toward osteogenesis [49–51]. We observed that human MSCs cultured on 12 and 21 nm high nanoislands displayed greater mechanosensitivity to fluid shear compared with flat control, e.g., a greater number of cells responding in [Ca^2+^]_i_ under 5 dyne/cm^2^ fluid shear stress (Fig. 6). However, with increasing shear stress, overall level of Ca^2+^ sensitivity was increased (and potentially saturated) and nanotopography control became less significant. Our finding may suggest that specific scale nanotopographies could produce an optimal environment to promote stem cell mechanosensing activity. Considering that improving cellular reactivity to mechanical signals may be critically needed for successful regeneration of mechanically functional tissues (bone, cartilage, muscle, etc.), our data may suggest an improved insight into functional tissue engineering. Additionally, our data on [Ca^2+^]_i_ sensitivity under nanotopography-fluid shear has an analogy with [Ca^2+^]_i_ data in MFCs under nanofiber-stretch [37], as described in the previous section, in that specific nanotopography or nanofiber culture will affect Ca^2+^ mechanosensitivity in cells.

One recent study reported that MSC lineage specification could be governed by cellular contractile forces that are determined by topography-fluid shear cues [52]. They utilized both anisotropic (gratings) and isotropic (wells) topographies. Human MSCs seeded on 1 μm wells showed higher cell contractility, and displayed under fluid shear osteogenesis. On the other hand, MSCs seeded on 2 μm gratings had lower contractility and remained multipotent even under fluid shear stimulation. Related focal adhesion formation was also changed, e.g., MSCs seeded on wells had focal adhesions with increased area and number. With an inhibition of actomyosin, MSC differentiation was not detected regardless of topographic or fluid shear stimulation, suggesting the potential role of topography-flow-induced cellular contractility in MSC fate determination.

## Conclusions and perspective

All data taken together, cells may sense and respond to both substrate cues and mechanical stimuli in a simultaneous manner. Depending on the substrate cues, such as grooves and aligned nanofibers (anisotropic) or randomly/uniformly distributed topographic features (isotropic), cells display differential morphological adaptations (alignment, spreading, migration) and then altered downstream behaviors (growth, lineage commitment, differentiation). The studies highlighted in this article suggest a strong possibility that such cellular reactions to substrate cues could be modulated by external mechanical stimulations, stretch and fluid shear. Depending on the varying regimens of the mechanical stimuli (strain, shear stress, oscillatory or steady, etc.) and correlation with the substrate cue (e.g., direction/angle of stretch or flow), the mechanical stretch or fluid shear either synergistically or competitively regulated cellular responses. In addition to observations that cell-substrate interaction could be actively modulated by added mechanical stimuli, the integrative approaches using substrate-stretch and substrate-fluid shear will help correctly recapitulate complex cellular mechanosensing environments in vivo. This may thus provide significantly improved understanding of cellular mechanotransduction behaviors accounting biomimetic mechanophysical conditions.

On the other hand, with some limited number of reports on the substrate-mechanical integrative control, there still exist considerations to be addressed. First, a more extensive and systematic studies with using various substrate parameters and loading regimens are required. Currently, it is quite difficult to compare each data from different reports due to the wide varieties of substrate properties and loading conditions. The need becomes even more significant when considering the reports that the sensitivity of substrate-mechanical integrative control of cells may be highly dependent on the scale of substrate topographies and the level of mechanical forces from stretch and shear, as described above. Also, a consideration of the other loading mode, such as compression or impulsive pressurization, and the combinatory loadings thereof may help fully describe in vivo mechanical environments.

Technically, lacking information includes the exact quantification of the mechanical loading under the substrate-combined situations. For example, fluid shear will definitely alter from unperturbed laminar flows to more turbulent flows if applied on substrates with varying micro and nanotopographies. Also, depending on the properties of the topographic features (shape and modulus), local stain values at varying substrate topographic positons might be different to each other and from the apparently imposed macroscopic stains. Mechanical stretch of the substrates within the cell culture media will also give rise to fluid flows originally not planned. These changes have not been calculated yet, and their potential effects on cell behaviors not addressed either.

From the standpoints of mechanobiology and functional tissue engineering, perhaps the more important consideration may be how to regulate cellular mechanosensitivity in response to external mechanical loading. The topic of this review article, substrate-mechanical integrative control, may answer to the question. As hypothesized in our previous study [48], the question to be answered can be “Does specific substrate culture (topography, patterning, nanofiber, etc.) will increase cellular responsiveness to mechanical stimulations (stretch, fluid flow)?” and if so, “What are the specific substrate topographic/geometric cues or dimensions to induce such upregulation in cellular mechanosensing?” Furthermore, taking into account that conventional mechanotransduction pathway studies have only dealt with plain surface cultures, an important question will be “What are the molecular mechanosensors that govern the substrate-mechanical integrative control of cells?” Answering these questions will lead to a proper description of cells in vivo that are exposed to complex ECM-mechanical integrative conditions. This may then significantly help design advanced functional tissue engineering and regenerative medicine protocols.

## Abbreviations

[Ca^2+^]_i_: intracellular calcium concentration
ANF: atrial natriuretic factor
COX: cyclooxygenase
Cx43: connexin 43
ECM: extracellular matrix
MFC: meniscus fibrochondrocyte
MSC: mesenchymal stem cell
p-FAK: phosphorylated focal adhesion kinase
PGE2: prostaglandin E2
ROCK: RhoA kinase
siRNA: small interference RNA
TAZ: transcriptional co-activator with PDZ-binding motif
YAP: yes-associated protein
